# Error in Tables and eFigure

**DOI:** 10.1001/jamanetworkopen.2025.48957

**Published:** 2025-11-25

In the Original Investigation titled “Continuous Glucose Monitoring Frequency and Glycemic Control in People With Type 2 Diabetes,”^1^ published October 31, 2025, there were errors in Table 1, Table 2, and eFigure 2. In table 1, there were typos in the spacing of 3 values (eg, 2 756 instead of 2756). In Table 2, 1 value (−0.87) had a percentage sign (%) typo, and 2 values were listed as negative when they were positive values (0.29 in the range of basal insulin in the frequency 3 column and 0.14 in the basal insulin range for the control column). eFigure 2 was removed from citation in the article and removed from Supplement 1 because this information was presented in Figure 3. This article has been corrected.^1^
